# Antifungal Encapsulated into Ligand-Functionalized Nanoparticles with High Specificity for Macrophages

**DOI:** 10.3390/pharmaceutics14091932

**Published:** 2022-09-13

**Authors:** Susana P. Mejía, Daniela López, Luz Elena Cano, Tonny W. Naranjo, Jahir Orozco

**Affiliations:** 1 Max Planck Tandem Group in Nanobioengineering, Institute of Chemistry, Faculty of Natural and Exact Sciences, University of Antioquia, Complejo Ruta N, Calle 67 N° 52–20, Medellin 050010, Colombia; 2 Experimental and Medical Micology Group, Corporación para Investigaciones Biológicas (CIB), UdeA, UPB, UdeS, Medellin 050010, Colombia; 3 School of Health Sciences, Universidad Pontificia Bolivariana, Cl. 78b #72A-109, Medellin 050010, Colombia

**Keywords:** *Histoplasma capsulatum*, PLGA, Itraconazole, macrophage, functionalized nanoparticle, F4/80 receptor

## Abstract

Infectious diseases caused by intracellular microorganisms such as *Histoplasma capsulatum* represent a significant challenge worldwide. Drug encapsulation into functionalized nanoparticles (NPs) is a valuable alternative to improving drug solubility and bioavailability, preventing undesirable interactions and drug degradation, and reaching the specific therapeutic target with lower doses. This work reports on Itraconazole (ITZ) encapsulated into core-shell-like polymeric NPs and functionalized with anti-F4/80 antibodies for their targeted and controlled release into macrophages. Uptake assay on co-culture showed significant differences between the uptake of functionalized and bare NPs, higher with functionalized NPs. In vitro assays showed that F4/80-NPs with 0.007 µg/mL of encapsulated ITZ eliminated the *H. capsulatum* fungus in co-culture with macrophages effectively compared to the bare NPs, without any cytotoxic effect on macrophages after 24 h interaction. Furthermore, encapsulated ITZ modulated the gene expression of anti and pro-inflammatory cytokines (IL-1, INF-Y, IL-6 and IL-10) on macrophages. Additionally, the anti-F4/80 antibody-coating enhanced natural and adequate antifungal response in the cells, exerting a synergistic effect that prevented the growth of the fungus at the intracellular level. Functionalized NPs can potentially improve macrophage-targeted therapy, increasing NPs endocytosis and intracellular drug concentration.

## 1. Introduction

Nanoencapsulation is making its way into the administration of drugs and subsequent site-specific release at the target cell or tissue through active functionalization of NPs with proteins, antibodies, carbohydrates, and peptides, among other ligands [1]. Ligand-functionalized NPs enhance uptake extent and can considerably increase the stability and systemic bioavailability of the drug since it protects its physicochemical characteristics, avoids possible degradation before reaching the therapeutic target, can achieve adequate doses at the intracellular level and, for example, facilitate the elimination of pathogens. The release of adequate drugs at the intracellular level is a strategy to avoid generating drug resistance to pathogens such as fungus and prevent non-specific accumulation in other tissues while reducing their toxicity and adverse side effects [2,3]. These adverse effects are due to the drug’s action outside macrophages, the most important effector cells in host resistance, as in the case of histoplasmosis (HPM), used herein as a model of intracellular infection.

HPM is an endemic and systemic mycosis of primary pulmonary origin, affecting especially immunosuppressed and non-immunocompetent individuals [4]. HPM is caused by inhaling aerosols that contain the infecting particles (microconidia and small mycelial fragments) of the dimorphic fungus *Histoplasma capsulatum*. This mycosis has been reported in more than sixty countries on all continents, but it is endemic in the Americas [5]. The clinical presentation of HPM includes the acute, chronic pulmonary and progressive disseminated forms, the latter occurring especially in patients infected with HIV, considered of high prevalence with a 0.9% overall incidence of coinfection, reaching 27% in endemic areas and mortality rates up to 30% [6,7].

Although HPM can be treated with different antifungals, Itraconazole (ITZ) is considered the first choice to treat mild and moderate forms of HPM. Yet, at least twelve months of therapy is required for the acute and chronic forms and even longer times for the progressive disseminated form [8], causing adverse effects in patients. Hepatoxicity has been the most frequent failure, relevant for all antifungal agents, from mild anomalies in liver function to fatal fulminant liver failure [9,10]. Given orally, it causes nausea, vomiting, diarrhea, skin rashes, headache, etc. [11]. ITZ has other limitations such as high lipophilicity, low absorption capacity, low systemic bioavailability, high susceptibility of the active compound to gastrointestinal hydrolysis, and drug interactions, suggesting extreme caution when used as part of multiple drug therapy [12,13,14,15]. In addition, HPM treatments generally offer limited efficacy because many drugs degrade before reaching the target tissues or cells, hindering therapeutic levels [16,17,18]. As an alternative, Sporanox is the only commercial formulation of ITZ for oral administration, encapsulated with hydroxypropyl-β-cyclodextrin as an adjuvant that improves its solubility and biodistribution. However, it is contraindicated in patients with renal failure due to inefficient adjuvant removal [11].

It is then imperative to develop more efficient therapeutic strategies that shorten the treatment time and reduce the adverse side effects of drugs used to fight HPM. Different polymeric NPs have been reported in this context, searching for effective treatment with less toxicity and more patient-friendly regimes. These nanosystems have stability and high-load capacity and can transport one or more active principles (with similar or different physicochemical properties) or a combination of therapeutic and contrast image agents to be administered in the same formulation by various routes. Additionally, polymers can be chemically modified to achieve the optimal conditions required as active ingredient transport systems [19,20,21]. Among the most used, poly-(lactic-co-glycolic acid) (PLGA) is a highly biocompatible polymer approved by the Food and Drug Administration (FDA) that has a rate of adjustable biodegradation and modular mechanical properties [12].

Functionalization of the nanocarrier’s surface has been widely explored for diagnosis, drug delivery, and other biomedical applications. Different ligands can be attached to the NPs surface, depending on the expected effect and whether intended for one or more functions [22,23,24]. For example, ligands containing bulky hydrophobic molecules can be attached to nanomaterial surfaces to prevent core agglomeration or be coated with water-soluble polymers, such as polyethylene glycol (PEG), to increase solubility required in physiological conditions, stealth properties, and biocompatibility. Ligands may also be attached to the surface of NPs to define their properties or act as "tags" for their recognition by target cells, either infected host cells or the microorganism directly [12].

Clinically important antifungals such as clotrimazole and econazole have been nanoencapsulated to improve their oral bioavailability [25]. ITZ encapsulated in PEG-functionalized poly-lactic acid (PLA) NPs has much better biocompatibility than commercial ITZ-cyclodextrin formulations, increasing drug solubility and stability in a wide range of concentrations and pHs [26]. Additionally, in vitro evaluations of the PLA-PEG-ITZ complex showed sustained drug release and growth inhibition of *H. capsulatum* [16]. Highly hydrophobic amphotericin B (AmtB), used to treat leishmaniasis and fungal infections, is hampered by its high toxicity. Encapsulation of AmptB into chitosan and PLGA-based NPs has reduced the drug’s toxicity and increased the antiparasitic effect. Furthermore, AmtB-loaded NPs functionalized with mannan-type carbohydrates have shown to be an effective treatment against leishmaniasis. In addition to its antiparasitic effect, recognizing the carbohydrate by specific macrophage receptors causes an activation of the cell’s defense mechanisms, increasing the treatment effectiveness [27,28]. PLGA NPs loaded with Fluconazole and coated with the cationic polymer polyethyleneimine (PEI) (FLZ-NP-PEI) also improved antifungal activity against four strains of clinically relevant *Candida* spp. [29,30]. Those are examples of antimicrobials encapsulated into functional (or not) polymeric nanocarriers reported in the literature but not in connection to PLGA-based NP-encapsulated ITZ against HPM.

We report a therapeutic solution to fight HPM intracellular infection. The therapeutic uses ITZ encapsulated in PLGA NPs, which functionalized with anti-F4/80 antibodies demonstrated for the first time increased antifungal effect on murine macrophages infected with *H. capsulatum* compared with bare NPs. Overall, results pave the way to design highly efficient nanocarriers for drug delivery against intracellular infections. Current studies are focused on directing the ITZ-loaded NPs to the lungs as the target organ by pegylation of NPs to improve their stealth properties, testing other macrophage-specific ligands and intranasal administration routes that can help increase the amount of NPs in the target organ. Additionally, the immunomodulation effect of functionalized nanoparticles was studied in vivo.

## 2. Materials and Methods 

### 2.1. Reagents

Evonik (Essen, Germany) generously donated poly-lactic-co-glycolic acid (PLGA); LA: GA 75:25 (RG 752H) with 0.14–0.22 dl/g inherent viscosity (4 to 15 kDa). Sigma-Aldrich provided Nile Red (CAS 7385-67-3), Itraconazole (ITZ, CAS 84625-61-6), poloxamer 188 (CAS 9003-11-6, Kolliphor^®^), D-α-tocopherol polyethylene glycol 1000 succinate (vitamin E-TPGS, CAS 9002-96-4), voriconazole (CAS 137234-62-9), and phosphate-buffered saline solution (PBS D8573). MTT TOX-1 Kit, HMM Broth (F12 HAM nutrient medium, N6760), Janus Green (CAS 2869-83-2), sodium periodate (CAS 7790-28-5), Hoechst (CAS 23491-45-4), and TRIzol1 (Ref. 15596026) were purchased from Sigma-Aldrich (St. Louis, MO, USA). Fetal bovine serum (FBS) (Gibco, Ref. 16000044), Horse serum (HS) (Gibco, Ref. 16050122), Dulbecco’s Modified Eagle Medium (DMEM, Ref. 10569010), and the antibiotic penicillin-streptomycin (Ref. 15140122), DNase I (Ref. EN0521), 3,3′-dihexyloxacarbocyanine iodide (DIOC6, CAS 53213-82-4), and RT-qPCR kit (Ref. A46109) were from Gibco (Gibco, Thermo Fisher Scientific, Inc., Waltham, MA, USA). Acetonitrile (CAS 75-05-8), ethyl acetate (CAS 141-78-6), ethanol (CAS 64-17-5), and dimethylsulfoxide (DMSO, CAS 67-68-5) were purchased from Merck. Citric acid (CAS 77-92-9) and sucrose (CAS 57-50-1) were purchased from VWR Chemicals. Potassium chloride (CAS 7447-40-7), sodium chloride (CAS 7647-14-5), and monobasic potassium phosphate (CAS 7778-77-0) were provided by JT Baker (JT Baker, Thermo Fisher Scientific, Inc., Waltham, MA, USA). Sodium citrate dihydrate (CAS 6132-04-3) and dibasic sodium phosphate (CAS 7558-79-4) were purchased from Panreac. Anti-F4/80 (ab100790) and Alexa flour 488 (ab150077) were obtained from Abcam (Abcam, Cambridge, UK). Brain Heart Infusion Agar (BHI) was obtained from Difco Laboratories (Ref. 211065, Thermo Fisher Scientific, Inc., Waltham, MA, USA). 0.1 M citrate buffer pH 4.5–5.5, 50 mM MES buffer pH 6.0–6.5, 50 mM HEPES buffer pH 7.0, and 1 M PBS buffer pH 6.5 were prepared by dissolving the reagents as received in Milli-Q water 18 MΩ·cm filtered through a 0.2 µm membrane.

### 2.2. Fungal Growth Conditions

*Histoplasma capsulatum* isolated Hc 1980 was obtained from the collection of the Corporation for Biological Research (CIB), Medellín, Colombia. Yeasts were grown on BHI agar, supplemented with 5% sheep red blood cells, 0.1% L-cystine and 1% glucose at 37 °C with 5% CO_2_, and repot was performed every 5 days. Colonies were taken from the BHI culture and grown in a 50 mL conical tube with 10 mL of Ham’s F-12 (Sigma-Aldrich, St. Louis, MO, USA). The base composition of the HMM medium was supplemented with 18.2 g/L glucose, 1 g/L glutamic acid, 6 g/L HEPES, and 8.4 mg/L cysteine at 37 °C after shaking at 150 rpm (Innova^®^ 44, Thermo Fisher Scientific, Inc., Waltham, MA, USA), for 48 h to reach its exponential growth phase. The yeast suspension was passed 30 times through a 1 mL tuberculin syringe with a 27 G ½ needle to separate the aggregated fungal cells and isolate the individual yeasts. The suspension was left upright for 1 min and then 5 mL was taken from the top. Cell viability was determined with Janus Green B vital stain and cell suspensions were adjusted to the required value of yeast/mL according to the hemocytometer count.

### 2.3. Cell Line and Culture Conditions

The mouse peritoneal macrophage cell line J774A.1, AMJC2-11b THP-1, CHO, and C2C12 were obtained from the American Type Culture Collection (ATCC, Manassas, VA, USA). The cells J774A.1, AMJC2-11b, CHO, and C2C12 were cultured in DMEM, and the THP-1 in RPMI, cultured medium containing glucose and L-glutamine, was supplemented with 10% heat-inactivated FBS or HS and 1% penicillin/streptomycin to avoid contamination with bacteria. The cultures were kept at 37 °C in 5% CO_2_ to ensure a saturated humid atmosphere. The culture media were changed every 2–3 days and the cells were subcultured every time they reached 90% confluence.

### 2.4. Nanoparticle Assembly and Functionalization

NPs were prepared using a high-energy emulsification method using a PLGA polymer with a glycolic acid: lactic acid ratio 75:25 and a molecular weight of 7–14 kDa. Briefly, 75 mg of PLGA polymer and 6.6 mg of ITZ and 7.5 mg of tocopherol were dissolved in 5 mL of ethyl acetate (organic phase) and then poured 10 mL 1% Kolliphor^®^ P188 in citrate buffer pH 5.

The NPs were functionalized by covalent coupling of the antibodies using the carbodiimide method. First, the carboxyl groups from the outermost NPs surface were activated by reaction with 4:1 EDC (400 mM):NHS (100 mM), followed by the anti-F4/80 (ab100790) polyclonal antibody with a 1:10 antibody: NPs ratio in PBS pH 6.5 for 2 h at 4 °C [31]. The functionalized NPs were characterized by changes in the size and ζ-potential obtained by DLS and ELS. The measures were taken in a Zetasizer-Pro (Malvern Instruments, Malvern, UK), at 25 °C 160 after adequate aqueous dilution by triplicate. The NPs were formed and functionalized based on our recent report [32].

### 2.5. Detection of Antibodies on NPs

A total of 20 µg/mL of goat antiRabbit IgG Alexa Fluor 488 (AF 488) antibodies were added to the solution of functionalized NPs, and then 1X sterile PBS was added; this solution was incubated at room temperature for 30 min with constant shaking. Once the incubation was completed, it was brought to a final volume of 200 µL to measure by fluorescence spectrophotometry. A calibration curve with different concentrations of AF488 was made between 25 and 0.195 µg/mL, where the fluorescence intensity values were plotted against concentration, and the values of the unknown antibody concentrations were calculated from the straight-line equation.

### 2.6. Determination of the Minimum Inhibitory Concentration (MIC)

To evaluate the antifungal activity of ITZ encapsulated into functionalized NPs and the different NPs precursors and free ITZ against Hc CIB1980, the microdilution method for yeast (M27-A3) described by the National Committee of Clinical Laboratory Standards (NCCLS) was used [33]. Once the fungus growth was determined by visible turbidity with the help of an inverted mirror, the viability of the fungus was evaluated using the MTT method. The yeast suspension was prepared in a histoplasma-macrophage medium (HMM) and a 96-well Round-(U)-bottom plate. A total of 1.5 × 10^5^ yeast cells/well was added to 100 µL. The dilutions of each treatment were prepared in HMM medium with 0.5% DMSO adding 100 µL of the different treatments to each well at concentrations from 16 to 0.0009 µg/mL for a 200 µL/well final volume. However, empty NPs didn’t encapsulate ITZ, so we calculated the amount of NPs that we added when encapsulated ITZ. Therefore, concerning each ITZ concentration, the amount of NPs added was 222.2 to 0.027 µg/mL.

The medium with 0.5% DMSO without the drug and untreated yeast was used as a control. The plates were incubated at 37 °C for 7 days with aeration on a mechanical shaker at 150 rpm. After the incubation time, the MIC was defined as the lowest concentration of treatment that can inhibit macroscopic fungal growth and low viability in MTT. Based on this result, the mean inhibitory concentration (IC50) (i.e., the concentration required for both free ITZ and encapsulated ITZ to inhibit Hc growth by 50) was calculated. The tests were in triplicate.

### 2.7. Evaluation of the Cytotoxicity of the Nanobioconjugate on Macrophages

A total of 3 × 10^4^ macrophages J774A.1 or AMJC2-11b per well were adhered to a 96-well plate in 200 µL DMEM culture medium supplemented with 10% FBS for 17 h at 37 °C and 5% CO_2_. THP-1 monocytes were differentiated to macrophages adding 1.2 × 10^5^ cells per well and treated with 100 nM of PMA in 200 µL RPMI culture medium supplemented with 10% FBS and incubated 72 h at 37 °C, 5% CO_2_. Then 100 µL of empty NPs, bare-NPs or F4/80-NPs were added to make different concentrations between 0.125 to 0.007 µg/mL. However, empty NPs didn’t encapsulate ITZ, so we calculated the amount of NPs that would be added with encapsulated ITZ. Therefore, concerning each ITZ concentration, the amount of NPs added was 3.47 to 0.216 µg/mL. Each treatment was tested and incubated for 3, 5, 24, or 48 h at 37 °C, 5% CO_2_. After the respective incubation time, 20 µL of MTT was added and incubated for 2 h at 37 °C in 5% CO_2_. Then, 130 µL of solubilizer solution was added to each well to dissolve the formazan crystals, then incubated at room temperature in the dark overnight. The formazan crystal’s optical density (OD) was measured spectrophotometrically at 595 nm in a BioTek ELx808 ELISA plate reader (BioTek, Winooski, VT, USA). Control cells (no treated) were maintained in DMEM-GlutaMax medium supplemented with 10% FBS.

### 2.8. Study of the Antifungal Activity of the Nanobioconjugate in an In Vitro Cell Model

The assay was performed in 96-well plates (Falcon^®^); 3 × 10^4^ J774A.1 macrophages per well were adhered in 200 µL DMEM with 10% FBS for 17 h. Each macrophage monolayer was infected with 100 µL of yeast inoculum (1.5 × 10^5^ yeast) prepared in an unsupplemented DMEM medium (1: 5 macrophage: yeast ratio) and incubated for 3 h at 37 °C with 5% CO_2_. After, the non-phagocytosed yeasts were washed with PBS. Then, 0.031, 0.015, or 0.007 µg/mL free ITZ and encapsulated into NPs with and without functionalized anti-F4/80 antibodies were added and incubated for 6 h or 24 h. Also, empty NPs were added with the same conditions as a control [33]. However, we again calculated the amount of NPs we would add with encapsulated ITZ. Therefore, the amount of NPs added was 0.86, 0.43, and 0.216 µg/mL. After 6 h of incubation, the co-culture was washed twice with PBS to eliminate the non-endocytosed NPs and a new medium was added.

The plate was incubated again under the same conditions to complete 24 h. Then the supernatant was taken, diluted five times in PBS and seeded on supplemented BHI agar. On the other hand, 200 µL of cold, sterile water was added to each well to lyse the cells and recover the yeasts that the macrophages had phagocytosed. The lysate suspension was dissolved with PBS five times and plated on supplemented BHI agar. The BHI dishes with the lysates and supernatants of the co-cultures were incubated at 37 °C with 5% CO_2_ for 7 days. After the incubation time, (CFU) was counted. The experiments were performed in two independent trials in triplicate.

### 2.9. In Vitro Assay of Specificity 

A total of 3 × 10^4^ J774A.1 cells were adhered onto a 96 well plate in DMEM with 10% FBS for 16 h. After, the cells were infected with 100 µL of *H. capsulatum* yeast inoculum (1.5 × 10^5^ yeast) previously stained with calcofluor (100 µg/mL) and incubated for 3 h at 37 °C with 5% CO_2_. Then, 100 µg/mL of functionalized (or bare) NPs with encapsulated Nile red were added and incubated at 37 °C for 6 h with 5% CO_2_. Therefore, monolayers were washed three times with PBS to eliminate any endocytosed NPs. The cell cultures were characterized by fluorescence microscopy. Determination of Nile red inside the infected cells (area of Nile red) was measured indirectly to know the differences in ligands’ specificity. To corroborate the NPs uptake, we used DIOC dye to stain cells’ cytoplasm and localize the NPs and the fungus. The area of Nile red (%) was determined with ImageJ software version 64-bit Java 1.8.0 (National Institutes of Health, Bethesda, MD, USA). Fourteen images were captured with a 40X objective and analyzed randomly from different regions. Individual cells and Nile red areas were framed with the freehand selection to measure the inner region. These areas were taken as the total area, and the area of Nile red was calculated in Microsoft Excel 2016 (Redmond, WA, USA) package using the data.

Additionally, for verification of the intracellular localization of NPs, confocal laser scanning microscopy (cLSM) and flow cytometry were employed. The uptake of the NPs by the uninfected J774A.1 cells was quantified by flow cytometry after the treatment with 100 μg/mL for 3 h. The uptake was also visualized by confocal fluorescence microscopy after 3 h of the coincubation of the cells with a NPs suspension of 100 μg/mL. Details of these experiments are described in the Supporting Information. On the other hand, the specificity of NPs was evaluated in other types of cells, such as muscle cells and ovary cells (see supplementary material).

### 2.10. Gene Expression

Determination of cytokines in the co-culture of J774A.1 with *H. capsulatum* was evaluated after 6 h of incubation with 0.007 µg/mL ITZ encapsulated and functionalized (or not) with anti-F4/80 antibodies. Cells were removed from the culture plates and centrifuged at 3000 rpm at room T for 5 min. Total RNA was obtained using TRIzol1 and treating samples with DNase I. cDNA was synthesized using 500 ng of total RNA using the Maxima First Strand cDNA synthesis kit for RT-qPCR according to the manufacturer’s instructions followed by real-time PCR using Maxima SYBR Green/Fluorescein qPCR Master Mix (2X). The CFX96 Real-Time PCR Detection System (Bio-Rad, Headquarters Hercules, CA, USA) was employed to measure gene expression levels. Analysis of the melting curve eliminated the possibility of non-specific amplification or primer-dimer formation. Validation of housekeeping genes for normalization of mRNA expression was performed before gene expression analysis. Validation of control genes for normalization of mRNA expression was performed before gene expression analysis. The expression of genes related to an inflammatory response IL-1 β, IL-6, TNF-α and IFN-γ and anti-inflammatory IL-10 was evaluated. Measurement of messenger RNA (mRNA) expression was obtained by relative quantification comparing the expression level of the target gene relative to GAPDH (glyceraldehyde-3-phosphate dehydrogenase, housekeeping gene). The expression level was measured in triplicate. Primers sequences used were GADPH Fw: 5′- CATGGCCTTCCGTGTTCCTA-3′ Rv: 5′- GCGGCACGTCAGATCCA-3′, IL-1β Fw: 5′-CTTCAAATCTCGCAGCAGCACATC-3′, Rv: 5′-TCCACGGGAAAGACACAGGTAGC-3′, TNF-α Fw: 5′-GACAAGGCTGCCCCGACTACG Rv: 5′-CTTGGGGCAGGGGCTCTTGAC-3′, IFNγ Fw: 5′- GACATGAAAATCCTGCAGAGCCAG -3′ Rv: 5′- TCGCCTTGCTGTTGCTGAAGAAG -3′, IL-10 Fw: 5′-TGGGTTGCCAAGCCTTATCGG Rv: CTCACCCAGGGAATTCAAATGCTC-3′, IL-6 Fw: 5′-CAACCACGGCCTTCCCTACTTC Rv:TCTCATTTCCACGATTTCCCAGAG-3′ [34].

### 2.11. Statistical Analysis

GraphPad Prism software (version 8, San Diego, CA, USA) was used for all statistical analyzes; the normal distribution was determined by the Shapiro-Wilk test. Medians and interquartile ranges were used to analyze cytokine expression levels. Analysis of variance between multiple experimental groups was performed using the ANOVA test, and differences between groups were analyzed using Tukey’s test. *p* Values < 0.05 were considered significant. A *p*-value ≤ 0.01 was considered statistically significant.

## 3. Results

### 3.1. Functionalization of Nanocarriers

**Figure 1:**
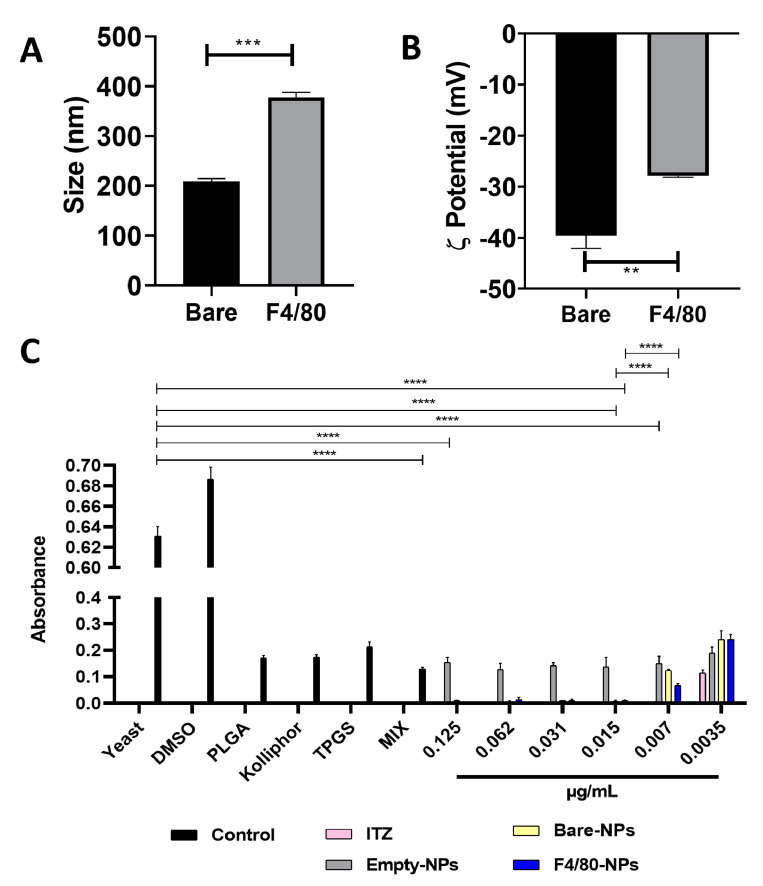
Comparison of size (**A**) and surface charge (**B**) of PLGA75: 25-TPGS-pH5-ITZ NPs functionalized or not (bare) with anti-F4/80 antibodies. (**C**) MIC strain *H. capsulatum* CIB1980 treated with free-ITZ, ITZ encapsulated into PLGA75:25 TPGS-pH5 NPs functionalized (or not) with anti-F4/80, empty PLGA75:25 TPGS-pH5 NPs and the corresponding amount of each NPs precursor (equivalent to the highest concentration of NPs that were used, i.e., 0.125 µg/mL). **, *** and **** indicate statistically significant differences with *p* < 0.001, *p* < 0.0001 and *p* < 0.00001, respectively.

The antibodies were coupled to the NPs by the carbodiimide chemistry. The functionalized NPs were characterized by changes in the size and ζ-potential by dynamic light scattering (DLS) and electrophoretic light scattering (ELS), respectively. The anti-F4/80 antibody-functionalized NPs presented a larger size, 226.66 ± 13.05 nm, than bare NPs 160.3 ± 9.5 nm (Figure 1A). ζ-potential showed a significant change, i.e., reduced the negative charge from −39.53 ± 2.54 mV to −27.9 ± 0.26 mV after covalent coupling with the antibody (Figure 1B). The antibody’s coupling was assessed by labeling it with the secondary antibody-Alexa fluor 488 and measured by fluorescence spectroscopy, obtaining 2.7% (0.059 µg antibody/mg of functionalized NPs); a similar result was reported previously [32].

### 3.2. Antifungal Activity

The minimum inhibitory concentration (MIC) of both free- and encapsulated-ITZ in the nanocarriers functionalized (or not) with anti-F4/80 antibodies was determined with a Colombian strain of *H. capsulatum* (CIB 1980). To determine the MIC, different concentrations ranging from 0.125 to 0.0018 µg/mL of free- and encapsulated-ITZ into functionalized (or not) PLGA75:25 TPGS-pH5 NPs were evaluated and empty NPs as a control. The macroscopic turbidity method and a 4,5-dimethylthiazol-2-yl) 2,5-diphenyltetrazolium bromide (MTT) assays (Figure 1C) showed a MIC down to 0.0035 μg/mL with free ITZ (*p* < 0.0001) and 0.007 μg/mL with both functionalized and ITZ-NPs (*p* < 0.0001), respectively. Empty-NPs in all concentrations and with all NPs precursors showed similar slightly inhibition-like fungus’ growth (*p* < 0.0001). Likewise, compared to the yeast control, a significant concentration-independent decrease was observed in the groups treated with empty NPs (*p* < 0.0001) and their precursors, indicating that these molecules could be inhibiting the fungus growth favoring the effect of the drug.

### 3.3. Cytotoxicity

**Figure 2:**
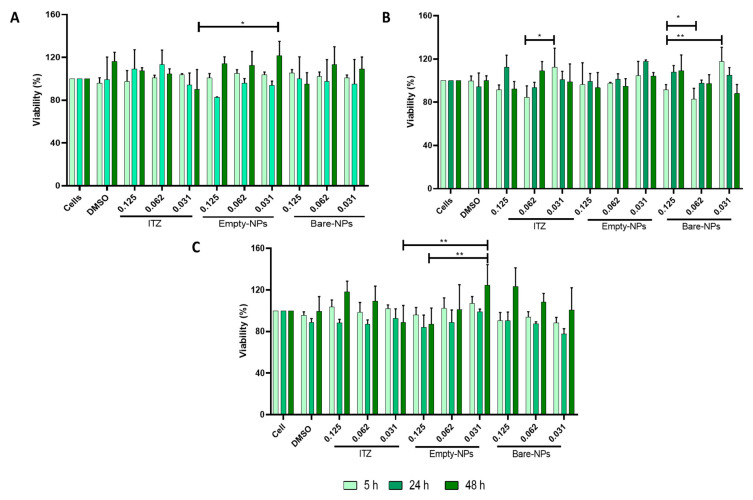
Cytotoxicity evaluation of ITZ encapsulated into PLGA75:25 TPGS-pH5 NPs by MTT with (**A**) J774A.1 (peritoneal mice macrophages), (**B**) AMJ2-C11 (alveolar mice macrophages), (**C**) THP-1 (human macrophages), with different concentrations and 0.125, 0.062, and 0.031 µg/mL ITZ for 5, 24, and 48 h, treatment with free ITZ and empty NPs and DMSO as controls. *, and ** indicate statistically significant differences with *p* < 0.01, and *p* < 0.001, respectively.

**Figure 3:**
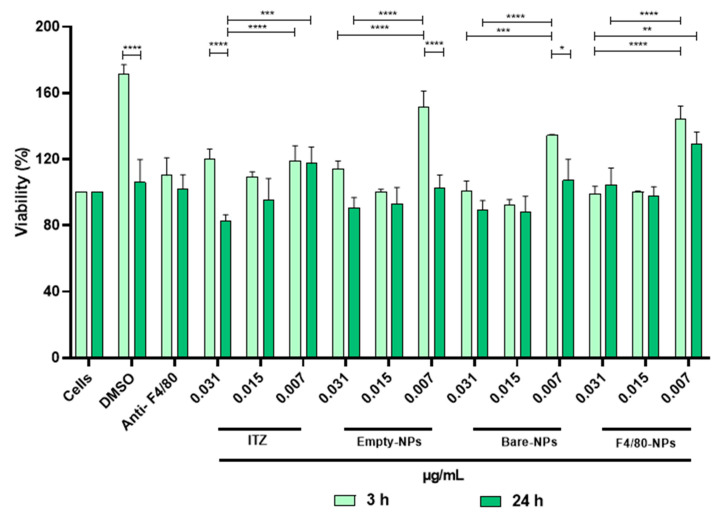
Cytotoxicity evaluation of ITZ encapsulated into functionalized PLGA75:25 TPGS-pH5 NPs with anti-F4/80 antibody on J774A.1 (peritoneal mice macrophages), with 0.031, 0.015, and 0.007 µg/mL of ITZ for 3 and 24 h, respectively by MTT and treatment with free ITZ and empty NPs or DMSO as controls. *, **, *** and **** indicate statistically significant differences with *p* < 0.01, *p* < 0.001, *p* < 0.0001, and *p* < 0.0001, respectively.

The cell survival plot for THP-1, J774A.1 and AMJC2-11b (Figure 2A–C) after 48 h of treatment with ITZ-NPs and empty NPs showed no reducing cell proliferation, with cellular viability higher than 80%. However, the viability after 5 h in AMJ2-C11 decreased depending on the treatment concentration with the bare NPs and free ITZ. Then, the cytotoxic effects of functionalized NPs in J774A.1 were evaluated, and a cell line was chosen to do the co-culture assay. Also, different times used to treat the co-culture (3 and 24 h) and higher, and lower NPs concentrations between the MIC concentration of functionalized NPs (0.062–0.007 µg/mL) were analyzed. Cell viability in all concentrations and with all types of NPs after 3 and 24 h of treatment was higher than 80% (Figure 3). However, the highest concentration of all treatments (0.031 µg/mL) significantly decreased cell viability compared to the lowest concentration (0.007 µg/mL) at both times evaluated (3 h and 24 h). Furthermore, when cells were treated for 24 h with the different formulations, a significant decrease in cell viability was observed compared to cells treated for 3 h, regardless of the concentration used. These results suggest that free ITZ and/or encapsulated into NPs and coated with F4/80 did not affect the viability of J774A.1 cells.

### 3.4. Antifungal Effect

**Figure 4:**
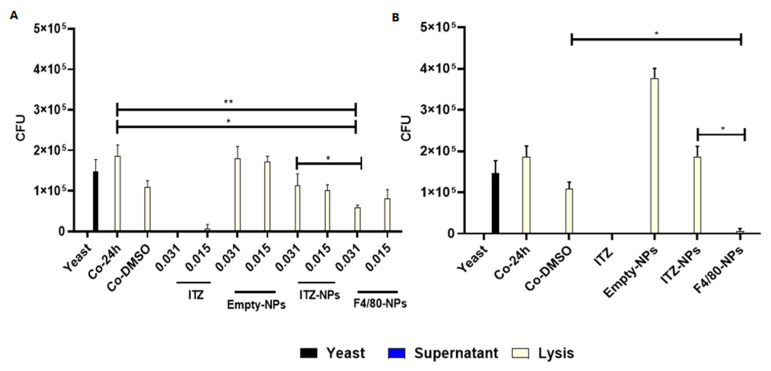
The efficiency of ITZ encapsulated into PLGA75:25-TPGS-pH5 NPs functionalized with the anti-F4/80 antibody to eliminate *H. capsulatum* yeast in co-culture (Co) with (**A**) J774A.1 by CFU counting of phagocytosed (lysed) or non-phagocytic yeasts (supernatant) after the treatment with 0.031 and 0.015 µg/mL of NPs with or without ITZ and free ITZ for 24 h (**B**) J774A.1 CFU counting of yeasts after the treatment with 0.007 µg/mL of NPs with or without ITZ and free ITZ for 6 h. *, and ** indicate statistically significant differences with *p* < 0.01, and *p* < 0.001, respectively.

The efficiency of ITZ encapsulated into PLGA75:25 TPGS-pH5 NPs to eliminate *H. capsulatum* in infected macrophages was evaluated by counting Colony Forming Units (CFU). For this purpose, co-culture of mouse macrophages J774A.1 (3 × 10^4^ cell/well, 96-well microplates) with *H. capsulatum* yeasts (1.5 × 10^5^ yeast/well, strain CIB 1980) were incubated under physiology conditions (37 °C with 5% of CO_2_) for 3 h. Then, the co-cultures were washed with PBS to eliminate the yeasts that were not phagocytosed and treated with 0.031, 0.015, or 0.007 µg/mL of NPs with free- and encapsulated-ITZ and empty NPs, and incubated at 37 °C during 24 h (Figure 4A) or 6 h (Figure 4B). The co-culture with 6 h of treatment was washed to eliminate the not endocytosed NPs and incubated at 37 °C to complete 24 h. Each well was aspirated and washed with PBS when the incubation was completed. The stock solution of 1:10 dilutions of not phagocytosed (supernatant) and phagocytosed (lysed macrophages) yeasts were plated (10 or 20 µL, respectively) onto BHI supplemented agar, and the CFU values were counted after 7 days of culturing.

Figure 4A shows that after 24 h with 0.031 and 0.015 µg/mL of ITZ encapsulated into NPs with F4/80, the CFU number from lysed macrophages significantly decreased compared to the control groups of untreated co-cultures and those treated with DMSO. Additionally, F4/80-NPs at a concentration of 0.031 µg/mL showed a significant reduction in the number of CFU compared to the values of bare NPs (*p* < 0.01). However, it is important to mention that the group treated with free ITZ significantly decreased CFU at all concentrations evaluated (0.031, 0.015, and 0.007 µg/mL) (*p* < 0.0001). On the other hand, in the lysates of the co-cultures washed after 6 h of treatment (Figure 4B), a significant reduction (*p* < 0.0001) was observed in the number of CFU obtained in the group treated with F4/80-NPs (0.007 µg/mL) in comparison with the control groups (co-cultures without treatment and treated with DMSO) and the group treated with bare-NPs. On the other hand, empty NPs- in co-culture did not show an antifungal effect (Figure 4A,B).

### 3.5. Specificity of Functionalized NPs for Macrophages

**Figure 5:**
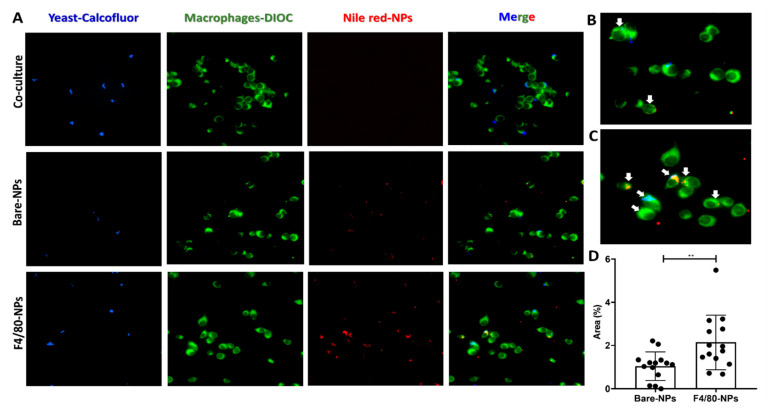
Endocytosis evaluation by fluorescence microscopy of anti-F4/80 antibody-functionalized PLGA75:25-TPGS-pH5-NPs encapsulating Nile red into J774A.1 macrophage *H. capsulatum* co-culture. (**A**) Images corresponding to the co-culture without NPs, bare-NPs, and F4/80-coated-NPs upon 6 h of incubation. Images from left to right were taken with DAPI filter (yeast-calcofluor stain), DIOC filter (macrophages-DIOC stain), TRITC filter (Nile red-encapsulated NPs), and merged (right), respectively. The scale is 20 μm. The zoomed-in (**B**) bare-NPs merged image and (**C**) F4/80-coated-NPs merged image. White arrows indicate macrophages infected with *H. capsulatum* with endocytosed NPs. (**D**) Endocyted NPs estimated by measuring Nile red by fluorescence intensity as described in the materials and methods section. ** indicates statistically significant differences with *p* < 0.001.

Nile red was used here as a model of a hydrophobic compound to evaluate the functionalized NPs specificity for J774A.1 macrophages infected with *H. capsulatum*. Fluorescence microscopy evaluated the intracellular co-localization of F4/80-coated-NPs and the fungus into the macrophages. The merge images in Figure 5A(down),C show that anti-F4/80-antibodies increased NPs endocytosis (Nile red-NP, blue fungus yeast and green cytoplasm cells), concerning bare-NPs that showed lower uptake by macrophages (Figure 5A(middle),B). Nile red fluorescence was estimated as described in the materials and methods section to confirm the uptake differences among the functionalized (or not) NPs. In this fashion, the macrophage’s uptake extent was twice for those treated with F4/80-coated-NPs (2.14%) than those treated with bare-NPs (1.04%) (Figure 5C).

To confirm the uptake of nanoparticles into macrophages, we performed two additional confirmatory experiments with different methodologies, such as flow cytometry and confocal laser scanning microscopy (cLSM) (Figures S1 and S2). J774A.1 macrophages were treated with 100 µg/mL of NPs with encapsulated Nile red, functionalized (or bare) and incubated for 3 h. After 3 h, both flow cytometry and (cLSM) showed significant differences between the uptake of functionalized and bare NPs, higher with functionalized NPs (Figure S1, *p* < 0.01 and Figure S2, *p* < 0.001, respectively). Additionally, the cLSM images showed that the NPs were located in the cytoplasm and not in the membrane (the merged image didn’t show yellow color) (Figure S2).

On the other hand, to corroborate the specificity of nanoconjugates C2Cl2 mice muscle cells and CHO hamster ovary cells were treated with F4/80-coated-NPs and bare-NPs for 3 h (Figures S3 and S4). Both cells did not show any NPs uptake. 

### 3.6. Immunomodulation

**Figure 6:**
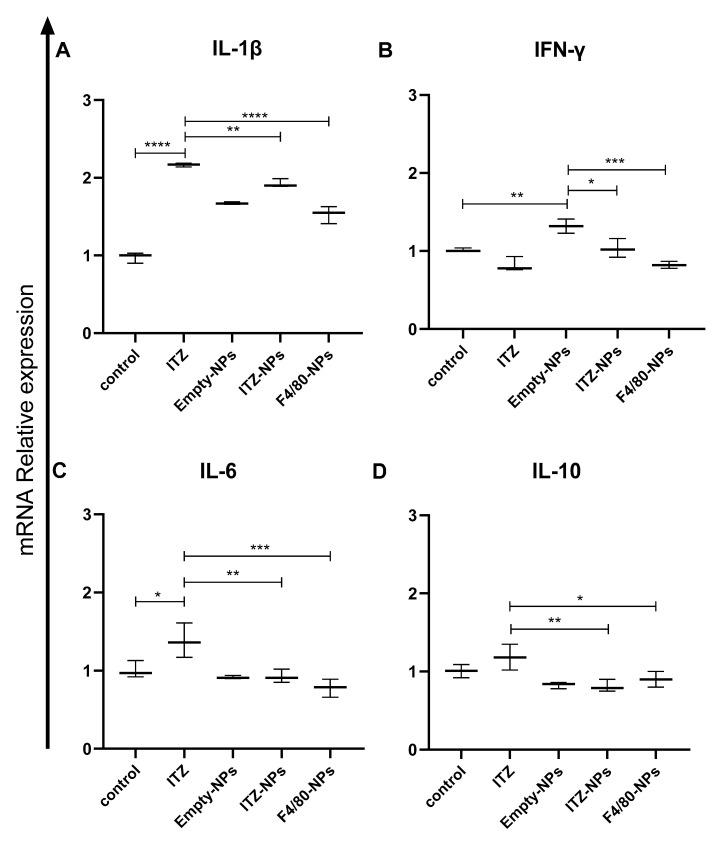
Immunomodulation effect of ITZ encapsulated into anti-F4/80-antibody-functionalized PLGA75:25-TPGS-pH5 NPs in co-culture of J774A.1 with *H. capsulatum* after 6 h of treatment. Expression of (**A**) IL-1β, (**B**) IFN-γ, (**C**) IL-6, and (**D**) IL-10. *, **, *** and **** indicate statistically significant differences with *p* < 0.01, *p* < 0.001, *p* < 0.0001, and *p* < 0.0001, respectively.

Immunomodulation is understood as the modulation or modification of the immune response, not strictly of the immune system, referring to a living being. Therefore, different biomolecules can modulate the immune response [35]. The capacity of functionalized (or not) ITZ-encapsulated nanocarriers to modulate or modify the immune response in macrophages infected with *H. capsulatum* after 6 h of treatment at concentrations that presented an antifungal effect (0.007 µg/mL) was studied by analyzing the expression of the pro-inflammatory (IL-1β, IL-6, TNF-α, IFN-γ) and anti-inflammatory cytokines (IL-10) at the RNA level (Figure 6A–D). Results indicated that ITZ treatment significantly increased IL-1β expression (*p* < 0.0001) compared to co-cultures without treatment (Figure 6A). However, IL-1 expression was reduced in the presence of ITZ nanoformulations, being more significant in the presence of F4/80-coated-NPs (Figure 6A). In the case of INF-γ (Figure 6B), empty-NPs significantly increased cytokine expression (*p* < 0.0001). In contrast, the levels of INF-γ significantly decreased with bare-NPs and F4/80-coated-NPs, but this decrease is more significant with the functionalized NPs treatment (*p* < 0.001). Additionally, it was observed that, compared to untreated co-cultures, treatment with free ITZ significantly increased IL-6 expression (*p* < 0.01) (Figure 5C). Contrary, treatments with ITZ nanoformulations (bare-NPs and F4/80-coated-NPs) significantly decreased the expression of IL-6 compared to free ITZ (Figure 6C). On the other hand, no statistically significant differences were observed between the different treatments and the expression of TNF-α. 

Furthermore, the co-cultures treated with the different nanoformulations (empty-, bare- and F4/80-coated NPs) significantly decreased the expression of IL-10 anti-inflammatory cytokine (*p* < 0.001) compared to the group without treatment.

## 4. Discussion

Intracellular microorganisms cause a wide range of infectious diseases with different degrees of clinical severity, representing significant morbidity and mortality worldwide [36]. Currently, infections caused by facultative intracellular microorganisms such as *H. capsulatum* are a great challenge because they have a more varied relationship with their hosts; the intracellular localization of the fungus makes it difficult to treat the infection, as macrophages can act as a barrier, preventing the antifungal drug from interacting with its target into the cell [37,38].

Fungal infections are often defined as challenging to treat due to the few options on the market and the multiple resistance that microorganisms are generating to existing treatments, the toxicity of antifungals, and their interaction with other drugs [39,40]. According to data reported by the FDA, since 1983, the generation of new drugs to fight infectious diseases, in general, has decreased, leaving us with fewer options. It is then imperative to develop a new efficient therapeutic strategy to improve the treatment time and reduce the adverse side effects and general issues to fight HPM. Encapsulation of microbial agents into NPs to selectively target pathogens has been shown to improve the efficacy and efficiency of therapeutic regimens. Thus, it is a strategy to overcome the challenges of conventional drug delivery systems that may actively drive drugs for site-specific and efficient drug uptaking, thereby readily reaching therapeutic intracellular levels [31,41,42,43,44,45]. Several NPs have been tested as potential drug delivery systems, including biodegradable polymeric NPs [1].

In this study, antifungal ITZ was encapsulated into PLGA NPs and further functionalized with anti-F4/80 antibodies, demonstrating an increased antifungal effect on murine macrophages infected with *H. capsulatum* compared with bare NPs. Increased size (Figure 1A) and decreased negative ζ-potential (Figure 1B) of the NPs suggest the antibody-NP surface binding without compromising its stability in maintaining a negative ζ-potential -29 mV (Figure 1B), avoiding NPs aggregation and remaining as a monodisperse colloidal system as reported [32]. Additionally, size remains very close to 200 nm (Figure 1A), which is essential to reduce the risk of rapid elimination by the endothelial reticulum system (RES), one of the main physiological barriers faced by NPs, affecting their biodistribution. Furthermore, several reports have shown that modifying the size and surface charge can regulate the interaction with physiological proteins (protein crown), modulating the type and quantity of proteins that bind to the surface; thus, recognition of NPs by specific receptors, facilitating (or not) the NPs uptake by the macrophages [12,46,47]. Therefore, the importance of functionalizing NPs with ligands for targeting cells and/or pegylation to neutralize the NPs surface charge and provide hydrophilic properties or steric hindrance stabilization, reduce the plasma proteins’ adherence and unspecific phagocytosis, and increase the specificity of targeting ligands [1,12]. By evaluating MICs with free- and encapsulated ITZ with (or without) anti-F4/80 antibodies, it was possible to show that encapsulated ITZ into functionalized NPs preserved its antifungal activity against the fungus. Encapsulated ITZ required a similar concentration (0.015 µg/mL) concerning bare NPs but a higher concentration to free ITZ (0.007 µg/mL) (Figure 1C). The MIC difference between NPs and free ITZ is related to the type of kinetic release that the NPs presented [32], where after 7 h incubation, the NPs achieved a release of 40% of the encapsulated ITZ and the release continued sustainable until 72 h, achieving only 43% [32]. Therefore, to inhibit 100% of the fungus growth, more concentration of encapsulated ITZ would be necessary than the free one. Nevertheless, the diffusion of more ITZ from the hydrophobic NPs core to the hydrophilic phase is hindered because the concentration of ITZ in the assay with NPs may be achieving the maximum saturation point. Furthermore, the NPs components, considering the controls’ results, didn’t show any inhibition. On the contrary, the empty NPs and each of the components of the NPs (0.125 µg/mL) showed a slight inhibitory action against the fungal growth. The inhibition may be explained because a high concentration of NPs or their components binds easier to fungus molecules, including proteins, affecting their growth [32].

In vitro model, the PLGA75:25-TPGS-pH5 NPs loading with ITZ showed an initial burst release followed by a slower and more persistent release [32]. The degradation of PLGA-NPs in vitro is caused through a bulk erosion mechanism [48], which consists of three phases. In the first phase, the release is due to the cleavage of the ester bonds in the polymer, decreasing their molecular weight without loss of formulation mass. In the second phase, the polymer loses mass because of the microenvironment acidification, forming oligomers. Finally, the solubilization of the polymer is caused due to the fragmentation of oligomers in monomers [49]. However, in vivo model, the release of ITZ from the nanoparticles could be 100%. This fact is due to the degradation of the ester linkages of the polymer occurring acceleratedly because the tissue cells recognize the NPs as foreign agents, producing enzymes and free radicals that can promote the degradation of PLGA. On the other hand, PLGA oligomers in the blood are more soluble compared to the in vitro medium [50]. Additionally, the ITZ encapsulated in the in vivo model could produce a higher effect than free ITZ due to the nanosystem protecting the degradation of ITZ at low pH. Some reports showed the activity of ITZ reduced under pH 5, being the MIC higher than the MIC under pH 7 [51].

When evaluating cytotoxicity, bare- and empty-NPs did not affect the viability in human and mice macrophages at different concentrations (Figure 2A,B). The NPs material precursors are one of the main factors affecting NPs toxicity. The NPs evaluated in this study mainly consist of the PLGA polymer with low toxicity due to their high degree of biodegradability, biocompatibility, and stability under physiological conditions. PLGA is biodegraded to lactic and glycolic acid in the physiological environment by hydrolysis of its ester bonds, which can be further metabolized through the Krebs cycle in carbon dioxide and water. Therefore, different pharmaceutical products based on this polymer currently exist with a long history of clinical use [12,52,53,54,55,56]. Moreover, functionalized NPs did not show a cytotoxic effect, suggesting that the antibody didn’t induce the wrong activation of cells. The results confirm the safe use of NPs in vitro and in vivo models (Figure 3).

The UFC assay showed the effectiveness of functionalized NPs in macrophages infected with *H. capsulatum* (Figure 4A,B). The functionalized NPs reduced the amount of UFC significantly concerning bare-NPs at a lower concentration of 0.007 µg/mL. This effect might be explained due to the significant increase of endocytosis in the macrophages of functionalized NPs, as we reported elsewhere [32] and also demonstrated by flow cytometry and the cLSM assay in this study (Figures S1 and S2). Furthermore, fluorescence microscopy images (Figure 5A,B) and the Nile red area quantification (Figure 5C) showed more macrophages with intracellular co-location of NPs and fungus. Therefore, we demonstrated that before 6 h of incubation, the NPs cell uptake was enough to get an antifungal effect compared with the bare-NPs even after 24 h (Figure 4B). Different types of macrophages, which are characterized by their heterogeneity, plasticity, and the type and amount of diverse receptors, are known to exist. In this sense, in mice, peritoneal macrophages present an intermediate expression of F4/80, low expression of mannose, and no expression of siglec-F receptor compared with lung macrophages [57].

It has been reported that the ability of NPs to modulate the immune response is a product of their physicochemical properties (size, charge, structure, composition, etc.) Other factors that also influence it are modifications in its surface and the therapeutic load [12,58,59]. The immune system cells recognize many of the components of NPs as foreign, triggering different immune responses through a complex process. Therefore, subsequent experiments aimed to determine analysis whether the administration of NPs-F4/80 alters the expression of genes related to the inflammatory and anti-inflammatory response in J774A.1 macrophages infected with *H. capsulatum* yeasts by PCR and explore whether this effect could be used as a mechanism that acts synergistically with the drug and enhances the effect of the treatment.

Macrophages are considered the most important effector cells in host resistance against histoplasmosis, participating in innate and adaptive immunity. Macrophages express a variety of pattern recognition receptors (PRRs) on their surface, including toll-like receptors (TLRs) and C-type lectin receptors (CRLs) that recognize pathogen-associated molecular patterns (PAMPs) of *H. capsulatum* such as Hsp60, Yps3, nucleic acids, glucans, mannan, among others, promoting its phagocytosis and the production of cytokines [60].

These cells express TLR2, the first documented TLR to bind to *H. capsulatum* and trigger an immune response. Its ligand is the surface protein Yps3. After its recognition, the adapter protein MyD88 is activated, a link between the extracellular receptor and the internal signaling pathway that leads to the transcription factor NF-κB entering the nucleus and activating several innate immune response genes such as TNF-α and IFN-γ [61]. They also express Dectin-1, a member of the C-type lectin receptor (CRL), which specifically binds to β-glucans, one of the main components of the *H. capsulatum* cell wall and the most inflammatory [62]. Upon recognition of β-glucans, Dectin-1 mediates Sky-dependent NFAT and NF-κB pathways and Sky-independent Raf-1 pathways to promote cytokine and chemokine production.

Our results show that free ITZ in co-cultures significantly increased the expression of IL-1β (Figure 6A). Other studies have reported that ITZ may directly modulate the immune response [63,64,65]. Similar results have been reported in other models; in an in vitro sepsis model, co-stimulation of ITZ and lipopolysaccharides (LPS) increased gene expression levels of IL-1β [65]. LPS are an essential component of the outer membrane of Gram-negative bacteria and act as a potent inducer of pro-inflammatory responses in monocytes and macrophages [66]. Similarly, this study’s results indicated a co-stimulatory effect of *H. capsulatum* and ITZ (Figure 6A). It was also observed that when the co-cultures were treated with different nanoformulations, the expression of IL-1β was significantly reduced compared to free ITZ. It suggests that modulation of the expression of this cytokine may be more related to the nanocapsule than with direct activation by the anti-F4/80 antibody since empty and bare-NPs also showed a reduction of IL-1β expression (Figure 6A).

IFN-γ plays a central role in HPM control, being the crucial cytokine to initiate the effector phase of cell-mediated immunity by activating macrophages to enhance microbicidal activity [67]. The modulation of a pro-inflammatory immune response was shown to be beneficial in controlling intracellular pathogens [27]. Darwich L. et al. demonstrated in an in vitro model that macrophages derived from monocytes after treatment with IL-12 and IL-18 produced IFN-γ. Therefore, human macrophages and lymphoid cells contributed to the IFN-γ response, providing another link between innate and acquired immune responses [68]. In our model, we didn’t measure the expression of IL-12 and IL-18. However, some reports demonstrated that the peritoneal macrophages express IL-18 precursor and usually activate during infectious diseases because most pathogens trigger the synthesis of mature IL-1β, which, in turn, activates caspase-1 to cleave pro-IL-18 to mature IL-18, which is then secreted [69,70]. Additionally, some reports showed macrophages infected with *H. capsulatum* produce IL-12 in low concentrations [71]. In agreement, it was observed that *H. capsulatum* in the macrophages induced the expression of IFN-γ (Figure 6B). Interestingly, treatment with bare-NPs and nanoencapsulated ITZ induced a higher cytokine expression in co-cultures, demonstrating the immunomodulatory role ITZ can exert. The immunomodulation effect in both treatments (free ITZ and bare-NPs) might be related to the modulation of IL-12 and IL-18, but more study is necessary to demonstrate it. However, functionalized NPs reduced the expression of this cytokine, indicating the possible role of the anti-F4/80 antibody as a binding molecule with immunomodulatory properties (Figure 6B). Consistent with our results, Warschkau et al., in an in vitro model of Listeriosis, showed that incubation with an antibody directed against the murine macrophage surface glycoprotein F4/80 reduced IFN-γ expression levels [72]. The F4/80 antigen is widely used as a specific marker for macrophages. Macrophages from the spleen (red pulp), lung, liver, peritoneal cavity, and nervous system express the F4/80 receptor. The interpreted amino acid sequence indicates a seven-transmembrane molecule with homologies to human EMR1 and CD97. As in vivo F4/80 is downregulated following infection with *Bacillus Calmette-Guerin*, its expression depends on the activation state of macrophages [73]. However, no specific ligands or biological functions of the F4/80 antigen have been described [72].

Additionally, the results with bare-NP can be related to the phenomenon where the endocytosis of macrophages of foreign objects can be modulated by characteristics such as size, shape, surface charge, and hydrophobicity/hydrophilicity because they regulate the interaction with physiological proteins and the protein corona formation [74,75,76]. In our case, it was the proteins from the Fetal Bovine serum (FBS). The type of proteins composing the protein corona may affect on endocytosis pathway [12,77]. After the NPs endocytosis, it depends on which way the macrophages are activated and produce different cytokines (pro-inflammatory or anti-inflammatory) [78,79]. Acharya D. et al. observed significant upregulation of pro-inflammatory mediators during phagocytosis. Using qPCR, they determined that complement receptor-mediated phagocytosis increased levels of TNF-α, IL-1β, IL-6, and MMP-9, compared to FcγR-mediated phagocytosis and control unstimulated cells [79].

The Th17 response is important in controlling many fungal infections. However, during PMH, Th17 is beneficial but not essential to controlling the fungus [44]. Free ITZ treatment significantly increased IL-6 expression (Figure 6C). Interestingly, treatment with the ITZ nanoformulations (bare-NPs and F4/80-coated-NPs) significantly reduced IL-6 expression compared to free ITZ (Figure 6C). The foregoing indicates that the encapsulation of ITZ into NPs allows a sustained and controlled drug release so that the immunomodulatory properties of free ITZ are potentially mitigated with nanoencapsulation.

TNF-α is a protective cytokine that exerts multiple effects during *H. capsulatum* infection, including activation of phagocytic cells, induction of apoptosis, and control of the CD4+ phenotype [67]. Blockade of TNF-α has been shown to reduce nitric oxide production by macrophages during primary infection. Neutralization of TNF-α suppresses the ability of murine T cells to mediate protection against *H. capsulatum* [80,81]. No statistically significant differences were observed between the different treatments and the expression of TNF-α. These results indicate that the treatments evaluated would not affect the immune response of the macrophage concerning the expression of this cytokine.

The expression of genes encoding anti-inflammatory molecules such as IL-10 was also evaluated. Although it is a crucial cytokine in limiting excessive immune activation, IL-10 exacerbates infection by preventing the clearance of *H. capsulatum*. Furthermore, it negatively affected the development of the protective Th1 response [82]. ITZ administrated in co-culture produced a significantly higher expression of IL-10 (Figure 6D), confirming that *H. capsulatum* and ITZ could exert a co-stimulatory effect. In addition, it was observed that the treatment of co-cultures with the different nanoformulations (empty-, bare- and F4/80-coated NPs) significantly decreased the expression of IL-10 compared to untreated co-cultures and co-cultures treated with ITZ. This indicates that the encapsulation of ITZ in NPs allows a sustained and controlled release of the drug, modulating its effect on inducing this cytokine in macrophages (Figure 6D). This result suggests that the NPs evaluated here can modulate the anti-inflammatory immune response during HPM, associated with a decreased inflammatory response and potential infection control.

Overall, this study opens the door to implementing new therapeutic strategies using antifungal encapsulated into functionalized NPs capable of directing the drug towards cells or organs infected by *H. capsulatum,* modulating the immune response, thus enhancing the effect of the therapy. Yet, it is necessary to continue improving the formulation and getting better knowledge about immunomodulation from this kind of therapy; it is crucial to know the scope and actual benefit of this treatment.

## 5. Conclusions

The encapsulation of antifungals into functionalized nanocarriers offers an up-and-coming treatment alternative to enhance the antimicrobial effect by site-specific targeting drugs in complex sites where pathogens are harbored, potentially reducing the dose and the therapy time. Our study demonstrated the improved antifungal effect by reducing the amount of ITZ to control *H. capsulatum* in co-culture with peritoneal macrophages. This effect can be related to the encapsulated ITZ NPs, which can generate a modulation in the immune response and antibody-coating enhanced natural and adequate antifungal response in the cells, exerting a synergistic effect that prevents the growth of the fungus at the intracellular level. Yet, it is crucial to study how nanosystems may influence the immune response more in-depth. However, it is necessary to develop new studies focused on further evaluating how this type of NPs interacts with macrophages and induces the expression of genes related to the different immunological profiles, allowing a deeper understanding of the type of immune response being generated. Similarly, it would be valuable to evaluate in vivo the antifungal and immunomodulatory effects of NPs using the experimental model of histoplasmosis in mice.

## Data Availability

All data relevant to the publication are included.

## Supplementary Materials

The following supporting information can be downloaded at: https://www.mdpi.com/article/10.3390/pharmaceutics14091932/s1. Figure S1: Endocytosis evaluation of PLGA75:25-TPGS-pH5-NPs functionalized with (or without) anti-F4/80 antibody into J774A.1 macrophage by flow cytometry. Figure S2: Endocytosis evaluation of anti-F4/80 antibody-functionalized PLGA75:25-TPGS-pH5-NPs encapsulating Nile red into J774A.1 macrophage by confocal laser scanning microscopy. Figure S3: Endocytosis evaluation of anti-F4/80 antibody-functionalized PLGA75:25-TPGS-pH5-NPs encapsulating Nile red into C2C12 mice muscle cells by fluorescence microscopy. Figure S4: Endocytosis evaluation of anti-F4/80 antibody-functionalized PLGA75:25-TPGS-pH5-NPs encapsulating Nile red into CHO Hamster ovary cells by fluorescence microscopy.
