# Supplementary material for: Antifungal Encapsulated into Ligand-Functionalized Nanoparticles with High Specificity for Macrophages
Source: Pharmaceutics. 2022 Sep 13;14(9):1932. doi: 10.3390/pharmaceutics14091932 (PMC9501281; doi:10.3390/pharmaceutics14091932)
Supplement: Supplementary file 1 [file pharmaceutics-14-01932-s001.zip › pharmaceutics-1866386-supplementary.pdf]

pharmaceutics

Supplementary Material

Antifungal Encapsulated into Ligand-Functionalized Nanoparticles with High Specificity for Macrophages

Susana P. Mejía [1,2,†], Daniela López [2,†], Luz Elena Cano [2], Tonny W. Naranjo [2,3] and Jahir Orozco [1,*]

[1] Max Planck Tandem Group in Nanobioengineering, Institute of Chemistry, Faculty of Natural and Exact Sciences, University of Antioquia, Complejo Ruta N, Calle 67 N° 52–20, Medellin 050010, Colombia
[2] Experimental and Medical Micology Group, Corporación para Investigaciones Biológicas (CIB), UdeA, UPB, UdeS, Medellin 050010, Colombia
[3] School of Health Sciences, Universidad Pontificia Bolivariana, Cl. 78b #72A-109, Medellin 050010, Colombia
* Correspondence: grupotandem.nanobioe@udea.edu.co
† These authors contributed equally to this work.

Supplementary Material

Material and Methods

Cell uptake experiment by flow cytometry

1 × 10^5 J774A.1 cells have adhered onto a 24 well plate for 16 h at 37 °C and 5% CO_2. Then, 100 µg/mL of functionalized (or bare) NPs with encapsulated Nile red were added and incubated at 37 °C for 3 h with 5 % CO_2. Therefore, monolayers were washed three times with PBS to eliminate no endocytosed NPs. Then, the cells were detached by adding 250 µL versene buffer per well and 250 µL DMEM was added to stop the reaction. After, the detached cells were collected in 1.5 mL tubes centrifuged at 300 g for 5 min. Finally, the supernatant was discarded and the cell pellet was resuspended in 1 mL of PBS. Cellular uptake of the nanocarriers was quantified by flow cytometry analysis with an Attune NxT (Thermo Fisher, Bremen, Germany). For Nile red detection, a 501 nm excitation laser was employed with a 552/580 nm band-pass filter. Flow cytometry data analysis was conducted with Attune NxT Software (Thermo Fisher, Waltham, MA, USA)

Visualization of intracellular localization of NPs by confocal laser scanning microscopy (cLSM)

J774A.1 cells were seeded in 15 µ-Slide 8 well glass bottom (ibidi) with 5000 cells per well for 16 h at 37 °C and 5% CO_2. The cells were washed the next day, and 100 µg/mL of functionalized (or bare) NPs with encapsulated Nile red were added and incubated for 3 h with 5% CO_2. Images were acquired on an LSM SP5 STED Leica Laser Scanning Confocal Microscope (Leica, Wetzlar, Germany), composed of an inverse fluorescence microscope DMI 6000CS equipped with a multi-laser combination and an HCA PL APO CS2 63 × 1.2 water objective. The Nile red was excited at 561 nm and detected at 580–640 nm. Plasma membranes were stained with CellMask Green™ (1:1000 diluted, Thermo Fisher, Germany) 5 min before microscopy. Then CellMask green was excited at 514 nm and detected at 525–550 nm. The area of Nile red (%) was determined with ImageJ software version 1.53e (National Institutes of Health).

Four images were captured with a 40X objective and analyzed randomly from different regions. Individual cells and Nile red areas were framed with the freehand selection to measure the inner region. These areas were taken as the total area, and the area of Nile red was calculated in Microsoft Excel 2016 package using the data.

In vitro assay of specificity

3 × 10^4 C2C12 cells and CHO cell were adhered onto a 96 well plate for 16 h in DMEM with 10 % FBS and HS, respectively. Then, 100 µg/mL of functionalized (or bare) NPs with encapsulated Nile red were added and incubated at 37 °C for 3 h with 5 % CO_2. Therefore, monolayers were washed three times with PBS to eliminate no endocytosed NPs. The cell cultures were characterized by fluorescence microscopy. To corroborate the NPs uptake, we used DIOC dye to stain cells' cytoplasm and co-localize the NPs.

Additional results

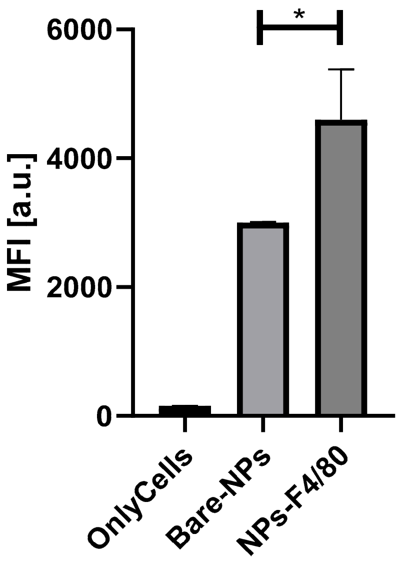

Figure S1. Endocytosis evaluation of PLGA75:25-TPGS-pH5-NPs functionalized with (or without) anti-F4/80 antibody into J774A.1 macrophage by flow cytometry. * indicates statistically significant differences with $p < 0.01$.

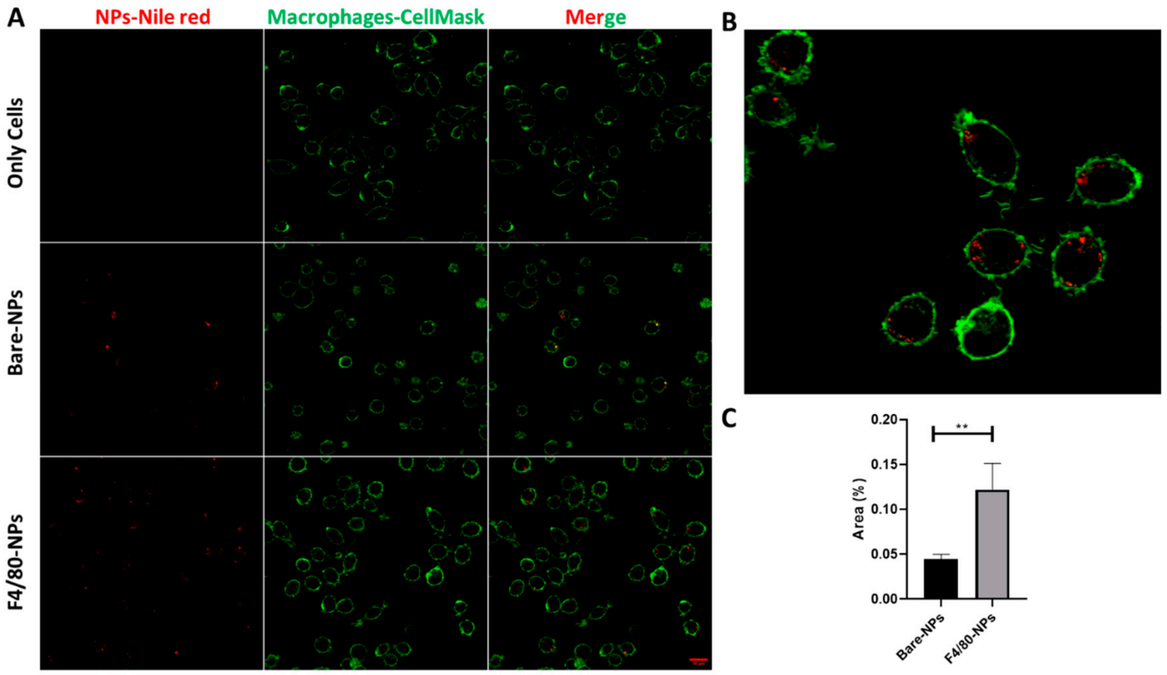

Figure S2. Endocytosis evaluation of anti-F4/80 antibody-functionalized PLGA75:25-TPGS-pH5-NPs encapsulating Nile red into J774A.1 macrophage by confocal laser scanning microscopy. (**A**) Images corresponding to the J774A.1 macrophage without NPs, bare-NPs and F4/80-coated-NPs upon 3 h of incubation. Images from left to right are Nile red-encapsulated NPs (left), macrophages-CellMask stain (center), and merged (right). The scale is 20 µm. The zoomed-in (**B**) F4/80-coated-NPs merged image. (**C**) Endocyted NPs estimated by measuring Nile red by fluorescence intensity as described in the materials and methods section. ** indicate statistically significant differences with $p < 0.001$.

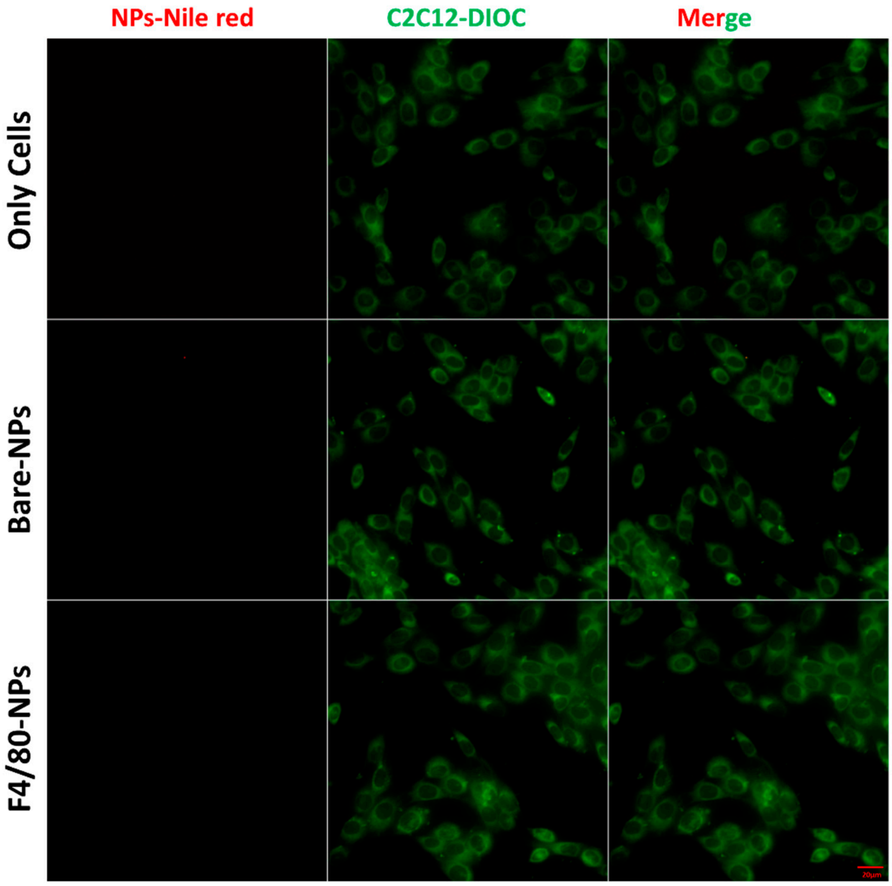

Figure S3. Endocytosis evaluation of anti-F4/80 antibody-functionalized PLGA75:25-TPGS-pH5-NPs encapsulating Nile red into C2C12 mice muscle cells by fluorescence microscopy. Images correspond to the C2C12 cells without NPs, bare-NPs, and F4/80-coated-NPs upon 3 h incubation. Images from left to right were taken with TRITC filter (Nile red-encapsulated NPs, left), DIOC filter (C2C12 cells-DIOC stain, center), and merged (right), respectively. The scale is 20 μm.

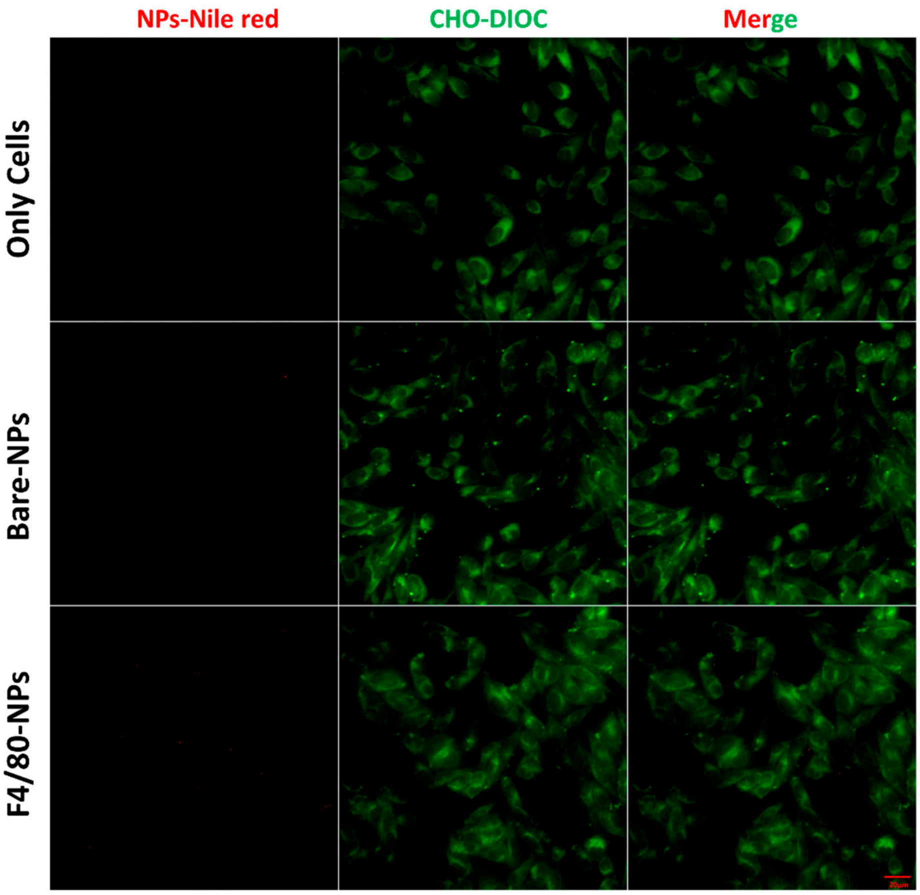

Figure S4. Endocytosis evaluation of anti-F4/80 antibody-functionalized PLGA75:25-TPGS-pH5-NPs encapsulating Nile red into CHO Hamster ovary cells by fluorescence microscopy. Images correspond to the CHO cells without NPs, bare-NPs, and F4/80-coated-NPs upon 3 h of incubation. Images from left to right were taken with TRITC filter (Nile red-encapsulated NPs, left), DIOC filter (CHO cells-DIOC stain, center), and merged (right), respectively. The scale is 20 μm.
